# Identifying perceived barriers to monitoring service quality among substance abuse treatment providers in South Africa

**DOI:** 10.1186/1471-244X-14-31

**Published:** 2014-02-05

**Authors:** Bronwyn Myers, Zainonisa Petersen, Rehana Kader, J Randy Koch, Ron Manderscheid, Rajen Govender, Charles DH Parry

**Affiliations:** 1 Alcohol and Drug Abuse Research Unit, Medical Research Council, Cape Town, South Africa; 2 Department of Psychiatry and Mental Health, University of Cape Town, Cape Town, South Africa; 3 Institute for Drug and Alcohol Studies, Virginia Commonwealth University, Richmond, Virginia, USA; 4 National Association of County Behavioral Health and Developmental Disability Directors, Washington DC, USA; 5 Department of Sociology and Centre for Social Science Research, University of Cape Town, Cape Town, South Africa; 6 Department of Psychiatry, Stellenbosch University, Cape Town, South Africa

**Keywords:** Performance measurement, Service monitoring, Service quality, Substance abuse treatment, South Africa

## Abstract

**Background:**

A performance measurement system is planned for South African substance abuse treatment services. Provider-level barriers to implementing these systems have been identified in the United States, but little is known about the nature of these barriers in South Africa. This study explored the willingness of South African substance abuse treatment providers’ to adopt a performance measurement system and perceived barriers to monitoring service quality that would need to be addressed during system development.

**Methods:**

Three focus group discussions were held with treatment providers from two of the nine provinces in South Africa. These providers represented the diverse spread of substance abuse treatment services available in the country. The final sample comprised 21 representatives from 12 treatment facilities: eight treatment centres in the Western Cape and four in KwaZulu-Natal. Content analysis was used to extract core themes from these discussions.

**Results:**

Participants identified barriers to the monitoring of service quality that included outdated modes of collecting data, personnel who were already burdened by paperwork, lack of time to collect data, and limited skills to analyse and interpret data. Participants recommended that developers engage with service providers in a participatory manner to ensure that service providers are invested in the proposed performance measurement system.

**Conclusion:**

Findings show that substance abuse treatment providers are willing to adopt a performance measurement system and highlight several barriers that need to be addressed during system development in order to enhance the likelihood that this system will be successfully implemented.

## Background

This paper describes perceived barriers to implementing a performance measurement system designed to monitor the quality of substance abuse treatment in South Africa. There is a high prevalence of substance use disorders in South Africa [1], with approximately 6% of the adult population meeting DSM-IV diagnostic criteria for a substance use disorder during the past year [1]. In South Africa, a broad range of substances are abused, with alcohol being the mostly commonly abuse substance, followed by cannabis, methamphetamine and other amphetamine type stimulants, heroin, and cocaine [2]. Although, when left untreated, substance use disorders are associated with high levels of morbidity and mortality [3], the South African treatment system has difficulty in responding effectively to these problems [4,5]. Several studies have reported that people with substance use disorders perceive available treatment services to be of poor quality and limited effectiveness [6-8]. These perceptions are cause for concern, since they influence people’s decisions about whether to seek treatment [5,9]. Concerns about the quality of substance abuse treatment services are not unique to South Africa as several other countries have reported concerns about the quality of substance abuse treatment [10,11].

In response, there have been efforts to develop and implement performance measurement systems for substance abuse treatment in several developed countries, including the United States (US) [12-16], the United Kingdom (UK) [17,18], and Canada [19] among others [20]. While all of these performance measurement systems collect data on a standardised set of indicators, some systems use treatment process indicators (such as treatment engagement, treatment completion, and waiting time for services) [13-15] while others use patient outcome data to measure performance [18]. Regardless of how performance is measured, these systems are valuable as they generate information that can be used to monitor the quality of substance abuse treatment, identify targets for quality improvement, and assess the impact of interventions designed to improve system performance [8,21,22]. Despite these potential benefits, South Africa has not yet developed a performance measurement system for monitoring the quality of substance abuse treatment [23]. Although the South African Community Epidemiology Network on Drug Use (SACENDU) Project routinely collects demographic and drug use data on patients admitted to substance abuse treatment, this system does not collect data on treatment process indicators that could be used to monitor services (such as percentage of people who complete treatment) or patient outcomes [23] and therefore cannot be used to monitor service quality. For these reasons, a performance measurement system needs to be developed that will enable the quality of South African substance abuse treatment services to be monitored.

However, the presence of such a system does not mean that it will be used as intended. In the US where performance measurement systems based on the Washington Circle measures [12,13] and the Health Effectiveness Data and Information Set (HEDIS) [14] measure performance on treatment process indicators such as (engagement in care), provider-level barriers to collecting and analysing performance data have contributed to considerable variation in the extent to which performance measurement systems are used [24-26]. Barriers identified include: inadequate infrastructure and resources to support the collection of data on treatment quality [27,28]; reliance on paper forms that are time-consuming to complete [28,29]; poorly developed electronic information systems and limited use of health information technology [27,29]; concerns about data safety, confidentiality, and the protection of patients’ privacy [27-29]; and lack of training in basic research methods that limits their ability to analyse and interpret data and lack of willingness to collect data [24,30].

While these studies provide insights into provider-level barriers to collecting data on substance abuse treatment quality in the US, it is unclear whether these findings can be extrapolated to a low-and middle-income country such as South Africa. South Africa has considerably less health infrastructure than the US, with significantly fewer health and social service providers and facilities per 100 000 patient population [31]. Although South African treatment providers are likely to report some barriers that are similar to those described by US providers, they may report specific barriers that are unique to the South African context. No previous studies have explored perceived barriers to the collection of data on substance abuse treatment quality in South Africa. This knowledge gap hampers the development of a performance measurement system that is responsive to the needs of and that corresponds with the resources available to South African treatment providers. This paper aims to address this gap by exploring perceived barriers to monitoring service quality among South African substance abuse treatment providers.

## Method

### Study design and sites

As part of a larger project to develop service quality measures for South African substance abuse treatment facilities, we conducted three focus groups with treatment providers. This formative phase of the project was designed within a social constructionist epistemology in that the questions contained in the focus group discussion guide was designed to lead the participants as little as possible, and the interchange between facilitators and the participants was considered as equally relevant to the data.

The Western Cape (WC) and KwaZulu-Natal (KZN) provinces were purposively selected as sites for this project as they serve diverse population groups with dissimilar patterns of drug use and have different types of treatment infrastructure. KZN is a mainly rural province with the highest unemployment rates in the country [32]. In this province, the most common substances of abuse are alcohol and cannabis [2] and there are few treatment services [33]. In contrast, the WC is more urbanised with lower rates of unemployment [32]. However the WC has the highest prevalence of lifetime and past year substance use disorders in the country [1], with alcohol, cannabis, methamphetamine, and heroin being widely abused [2]. In comparison to other provinces, the WC is relatively well-serviced in terms of treatment facilities [33]. These two provinces, collectively, provided a good representation of the variety of service providers found nationally.

### Sample

Three focus groups were conducted: two groups (each consisting of eight participants) were conducted in the WC and one group (consisting of five participants) was conducted in KZN. We had initially planned to conduct more groups; however we reached saturation of new information after three groups and therefore decided to stop data collection at this point.

Sample selection comprised two steps. First, we purposively selected facilities from SACENDU’s database of treatment services in the WC and KZN. Maximum variation sampling techniques were used to ensure that the broad range of treatment facilities available in each province were represented (residential and outpatient, for-profit and non-profit services). Second, we purposively selected participants from these identified facilities to participate in the groups. To be eligible for inclusion, participants had to be permanently employed by the selected facility. Maximum variation sampling techniques were used to ensure that groups included participants with a diverse range of roles and responsibilities (such as addiction counsellors, social workers and service managers). The final sample comprised 21 representatives from 12 treatment facilities: eight treatment centres in the WC and four in KZN.

### Procedure

Project staff approached treatment facility managers in each province, described the study, and asked if they were willing for staff from their facilities to participate in a focus group discussion on the collection of data for monitoring treatment quality. If the facility manager granted this permission, we randomly selected one to two staff members from the list of staff at each facility to participate in the discussions. These selected staff were then approached and asked if they were willing to participate in the discussions. If they were unable to participate, we randomly selected another person from that organisation to replace them. This random selection ensured that we included participants with a variety of roles and responsibilities in the group discussions. All participants provided written informed consent prior to inclusion in the study. Focus groups were conducted between April 2011 and October 2011.

Focus group discussions were guided by a set of open-ended questions that were developed and reviewed locally by the project’s national steering committee that included representatives from several treatment facilities (see Additional file 1 for the semi-structured discussion guide). This discussion guide contained questions about the type of data currently collected by treatment services and how these data are used, their perceptions of barriers and facilitators to monitoring treatment services, their perceptions of the need for monitoring substance abuse treatment quality, perceived capacity to collect and use data on service quality, and their opinions about the proposed performance measurement system for South African substance abuse services. Each focus group discussion was facilitated by two of the South African co-investigators, both of whom are PhD graduates with more than five years of experience in conducting qualitative interviews and focus groups, and one of whom is a clinical psychologist with extensive experience in group dynamics. All groups were conducted in English, and the discussions were audio-recorded and transcribed verbatim. The duration of the discussions ranged between one and a half to two hours.

### Data analysis

Content analysis [34] was used to analyse these qualitative data. Due to the lack of data on performance measurement in South African substance abuse treatment services, the analysis was descriptive in nature. Focus group transcripts were first reviewed for emergent themes. After having identified emergent themes, these were then grouped into meaning units which were then assigned codes. Next, we grouped codes that were related to each other into preliminary categories. Finally, these categories were organised into overarching themes. We used Open-Code [35], a qualitative software programme, as a tool for coding the data. To establish inter-coder agreement, data were individually coded by two researchers (both of whom are PhD-level researchers in public health with more than five years of experience in coding qualitative data) who met to discuss coding procedures and definitions, compare notes and establish degree of agreement. Coding discrepancies were resolved by discussion. No new codes emerged after two-thirds of the transcripts were coded, indicating that we more than likely attained content saturation. Inter-coder reliability checks were conducted, with a Kappa score of 0.76 being obtained between the two coders.

### Ethics approval

The study received human subjects approval from the Centers for Disease Control and Prevention (CDC) and the University of Stellenbosch’s Health Research Ethics Committee.

## Results

### Sample characteristics

Of the 21 participants, seven (33.3%) were male and 14 (66.7%) were female. Participants’ ages ranged between 28 to 56 years old (mean age 41.7; SD 8.6). The sample comprised 11 social workers, four service managers, three clinical psychologists, two psychological counsellors and 1 medical doctor. In South Africa substance abuse treatment services are delivered predominantly by social workers [36]. Participants had been working in their respective positions between two and 20 years (mean 8.9; SD 6.4); and had been working in their respective professions between four and 30 years (mean 15.7; SD 7.9).

### Perceived barriers to monitoring service quality

The first overarching theme that emerged from the focus groups is “perceived barriers to monitoring service quality”. This theme refers to barriers to the collection and use of data for monitoring service quality. All participants noted that they put considerable effort into collecting patient data. While most participants felt that the data they collect are potentially useful, they reported several challenges to monitoring service quality. These challenges are described below.

#### Outdated methods of collecting data

Participants thought they collected valuable information from patients that could be used for monitoring service quality. However, they reported that their use of pen-and-paper modes of data capture and storage limited their ability to retrieve and use these data to assess service quality. While this issue was raised mainly by participants from publicly-funded non-profit treatment centres, some participants from private treatment centres also talked about the need to move away from hand-written notes and files to electronic information systems. Limited access to computers was cited as the main obstacle to migrating to electronic information systems. Only a handful of participants had access to computers and data entry software at their facility, as reflected in the following statements:

“Do you know what difference it would make if all our staff had access to computers, if all of us could enter our valuable client information on a PC (personal computer) and retrieve that information with a click of a button?” [FG2; female social worker]

“If only all our social workers had computers, or even just access to one computer only used by the social workers and psychologists, that would be the ideal…we can do more (data collection) if given the resources (computers and data entry software)”. [FG1; male service manager]

Participants ascribed many of the challenges they had in monitoring service quality to their overreliance on pen-and-paper data collection methods. One prominent example of this was the inconsistencies in the type and quality of data collected, largely because hand-written notes allowed for variation in terms of what content is recorded. All participants noted that the type of information recorded and the level of detail with which information is recorded varies by counsellor. Participants also reported that paper records are often not updated in a timely manner, there is often duplication of information recorded, there are difficulties in retrieving information from these files, and that files are sometimes misplaced. As one participant reflected:

“So we are really looking forward to a computerised system instead of the paper and pen so that things are captured and whoever is allowed access can have access to it immediately. The file won’t get held up somewhere or you lose it or just struggle to keep it together”. [FG1; female social worker]

#### The paper monster

All participants commented that they were overwhelmed by the amount of paperwork associated with current data collection practices. Participants were frustrated that facilities used very little of the information they collected and that there was duplication of information collected because of the use of multiple forms with overlapping content.

“We collect data from admission to discharge, which is important, but it often gets out of hand and I think the patients get tired of completing the same information over and over…and don’t you think it is unethical to bother them with forms and information that never even get used…” [FG1; male psychologist]

Several participants noted that completing this paperwork required a significant amount of time that could have been reserved for service delivery. This is reflected by the following comment:

“If you look at all the reports you have to write, you get to the stage where you write, write, write…., and in the end it’s about the client and not about writing. And now you are focused so on writing the reports, you are not getting to the client”. [FG2; female social worker]

Some participants reported that their facilities tried to reduce the amount of time clinical staff spent on paperwork through task-shifting data collection responsibilities to administrative staff. According to these participants, this was problematic as having multiple people responsible for data collection often diminished the quality of data collected. As one participant noted:

“We have so many people including data in the file: from the clerk, the administrator, the social workers, the psychologist…Sometimes data that should be collected by the social worker is collected by an admin person only to save time, and this creates a problem with missing information and accountability because you don’t know who does what”. [FG3; female social worker]

#### Overburdened staff with little time to collect data

Most participants reported that clinical staff in outpatient treatment settings have very high patient caseloads and are required to perform multiple functions. These overburdened personnel have little time to complete multiple and lengthy pen-and-paper forms. Almost all participants thought that expecting these overburdened staff to complete additional data collection forms would compromise data quality. They noted that overburdened personnel are less likely to collect reliable information. As one respondent reflected:

“There is so much our social workers have to do, so many assessments to complete, group notes, urine tests, and the list goes on…we don’t have someone dedicated to doing admissions and case histories and reports, so we really need to be careful about adding to their burden”. [FG2; female service manager]

#### Lack of skills to analyse and interpret data

Participants acknowledged that the forms they currently use to monitor service quality are potentially flawed. They recognised that poorly designed evaluation forms could lead to bias, limiting the usefulness of these findings for performance improvement efforts. However, they felt that they did not have the skills to improve these forms:

“We have our own evaluation forms, and patients often give us good reviews. We seldom have someone telling us that services are not good, but then we have so many patients not completing treatment. I think our evaluation forms need improvement and of course there is bias involved, but I do not know how to deal with that, we just do the best we can”. [FG 3; female social worker]

In addition, treatment service managers thought they lacked the skills to analyse and interpret their facility data which prevented them from using these data for service improvement efforts.

“One can often look at data and you don’t really know what it means”. [FG1; male service manager]

“We have very limited capacity, you know…to analyse data”. [FG2; female service manager]

“We can’t analyse it, if we want something complex answered we give it (the data) to someone else to do for us”. [FG3; male service manager]

On reflection, treatment personnel thought that they would be able to analyse and interpret their data if given the appropriate training, mentoring and support.

#### Between-facility variation in types of data collected

All participants identified considerable between-facility variation in the types of data collected and data collection formats used. Participants noted that most facilities had developed their own data collection forms, with facilities not collecting data on a shared set of indicators. Several participants raised questions about whether all treatment centres “are capturing the same kind of data”. Variation in the types of data collected and the manner in which data are recorded makes it difficult to compare facility performance on key indicators.

### Recommendations for improving the collection and use of service quality data

The second overarching theme was “recommendations for improving the collection and use of service quality data”. This theme refers to participants’ perceptions of possible solutions to these barriers. Participants provided several recommendations for how to address these perceived barriers to the collection and use of service quality data. Participants highlighted the adoption of contemporary data collection methods, the use of psychometrically validated measures, and the collection of service quality data using a shared set of common indicators as critical to enabling the monitoring of treatment quality.

#### Adoption of contemporary data collection methods

Participants emphasised the need to shift towards contemporary methods for data collection, storage and retrieval. Electronic health information systems (EHIS) were seen as a means to facilitate service monitoring, improve data quality and enhance the likelihood of data-driven quality improvement initiatives. This is reflected in the following statements:

“I worked in Ireland and they had that (electronic) system, and they did not have this paper trail there”. [FG2; female social worker]

“An electronic system would be very good. I mean like it would help standardisation of information taking”. [FG1; female addiction counsellor]

Participants also noted the potential benefits of having a central electronic patient information system in which people’s health, social welfare, criminal justice, and education records are contained within a single electronic system and accessible to any service provider on provision of the patient’s official identification number and the provider’s secure access code. Several participants believed that this would provide access to a wealth of information about the patient’s health and social status and limit the need for extensive history taking. According to participants this would streamline the data collection process:

“It kind of makes me think of the system they have in the UK…. the nice thing for me in the UK was that you have immediate access to a lot of different information about a client… Now at least if you have a system like that you know what’s happening with the client, you know where they are and that kind of stuff. You would be getting regular reports from teachers, police, different NGOs. So they would be sharing standardised information which would go into a central database. Anybody new who starts with the case would immediately get a history of everything that is going on”. [FG1; male social worker]

Nevertheless, some participants were reluctant to use a centralised electronic patient record system due to data safety and confidentiality concerns. Specifically, they were worried about their patients being identified as drug users and how this might affect their access to other services. They also voiced concerns about who would have access to these records:

“There’s a lot of shame around addiction and a lot of the stuff is illegal. You’ll get little 13 year old boys coming and not sure if they’ve got a problem, parents bring them; you don’t want to put someone like that on some kind of list that might stick with them/him.” [FG1; female psychologist]

Furthermore, the vast majority of participants noted that the successful implementation of an EHIS would require various resource and skills deficits within treatment facilities to be addressed such as limited access to electronic equipment, low levels of computer literacy, and poor analytic skills. This is illustrated below:

“My staff are doing the best they can, given the circumstances…but I would say that our annual reports for example is not as detailed and correct as the treatment centres that have these facilities (computers and staff trained in data analysis)”. [FG2; female service manager]

“It’s not just the initial outlay; it’s also maintaining it, the IT support”. [FG2; female service manager]

“Even the new social workers who are newly qualified, that come out of some of the universities, are still not very comfortable using computers”. [FG3; male service manager]

#### Using reliable and valid tools for monitoring service quality

Although many participants expressed a sense of pride in their evaluation forms, they also noted that these forms have not been rigorously tested and validated. All participants expressed the “need for tried and tested tools” that would generate reliable data for service improvement. Providers thought that using psychometrically validated measures of service quality would improve their confidence in the data generated. As one provider noted:

“I designed our evaluation forms myself, and it’s a very detailed form. We have been using this form for about a year now, and one of our students will use the tool as her master’s project, you know doing the cognitive testing and analysis. So as yet we are not sure whether it works…” [FG1; male psychologist]

#### Collecting service quality data on a shared set of indicators

Participants suggested that forms used to monitor service quality be standardised across facilities in order to allow for the collection of service quality data on a shared set of indicators. Participants believed that this would improve the quality of data collected and would allow the performance of treatment facilities to be compared on a common set of quality indicators. As participants reflected:

“If you want to determine the quality of the service that you give maybe that (data) can be collected in a standardised way”. [FG1; female social worker]

“There is an overlap across facilities; you find similar forms with similar information. We should be able to standardise, it’s difficult but we should”. [FG2; female social worker].

#### Willingness to participate in a performance measurement system for substance abuse treatment

All participants were supportive of an initiative to develop and implement a performance measurement system for South African substance abuse treatment services. Participants noted that the initiative could address many of the shortcomings of current data collection methods. Participants reflected that the success of this initiative would rest on the active participation of treatment providers in the design, testing and implementation of the system. They cautioned against imposing a performance measurement system on service providers without consultation:

“There are times when we don’t know what is important and what is of lesser importance, and we need help determining this. And I can tell you now already that people will not be happy to change what they are doing completely, unless what you are offering is better than our current efforts…the best way for you to know what will work at our centres is to involve us in the decision making else you will be met with a lot of resistance”. [FG3; male service manager]

## Discussion

South Africa invests a considerable amount of money in the treatment of substance use disorders, yet little is known about the quality and effectiveness of these services. Plans are underway to address this by developing a performance measurement system that is tailored to South African substance abuse treatment services [23]. In high-income countries, provider-level barriers have been shown to impact the use of these systems in both substance abuse treatment [25,26], mental health care [27,37], and general health care [38-40], however, until now, little has been known about the willingness of substance abuse treatment providers and their perceived challenges to monitoring treatment quality in low-and middle-income countries such as South Africa. Findings from this exploratory study show that while South African substance abuse treatment providers are willing to adopt a performance measurement system that enables the quality of services to be monitored, several provider-level barriers need to be addressed in order to ensure that this performance measurement system is feasible to implement and use.

More specifically, findings suggest that South African substance abuse treatment providers are prepared to implement a performance measurement system that will enable them to assess the quality of services they provide, even if this implementation compels them to change their current approaches to data collection. For instance, all participants stated that they would be prepared to adopt new scientifically robust tools to collect data on service quality. They acknowledged that they had limited confidence in the utility of their own evaluation forms and that adopting scientifically validated tools would increase their confidence in the data. Participants also saw benefits to ensuring that data were collected on a shared set of common indicators and in a uniform manner across facilities, including that they would be able to compare the performance of facilities on a shared set of indicators.

However, participants’ approval for the proposed performance measurement system was not without some caveats. Notable amongst these was a concern that new data collection processes would add to clinical staff’s already considerable paperwork burden. Providers commented that as clinical staff had high caseloads and were already overburdened with paperwork they may not be able to comply with the data collection requirements of the proposed system. This barrier to performance measurement is not unique to South Africa and has reported in studies on barriers to performance measurement in other settings [25-29] and for other areas of health care [40]. To address this concern, participants recommended minimising response burden by only collecting data on a minimum set of indicators and by streamlining current data collection processes to avoid the collection of duplicate and unnecessary information. Minimising response burden has been identified as an effective strategy for facilitating health care personnel’s support and commitment for performance measurement in other health care settings [37-40].

Participants also indicated that the pen-and-paper formats currently used to collect and store patient information not only made it difficult to standardise the type of information recorded but would also make it almost impossible to retrieve information that could be used for quality monitoring. Although participants recognised that an EHIS would address many of these problems and supported the idea of an EHIS, they cited resource limitations (lack of computers and technological support), overburdened staff, and computer literacy issues among staff as significant obstacles to the implementation of an EHIS. These impediments to EHIS have been experienced in other health and social welfare sectors in South Africa [36,41] as well as the US [25,27,37,40] and have resulted in incomplete and missing data [36]. Together these findings imply that for an EHIS to produce reliable data that can be used for performance measurement there will need to be considerable investment in health information technology and staff training within the substance abuse treatment sector. While this may raise questions about the extent to which an EHIS is feasible to implement in this low-resourced setting, there are several examples of EHIS that have been successfully implemented in other health services in sub-Saharan Africa using mobile technology [42,43]. Mobile health information systems are gaining popularity in African and other low-resourced regions because mobile technology is easily accessible and affordable and the technology is simple to use with limited training compared to traditional information technology platforms [43]. As South Africa has high levels of mobile phone coverage [43], this may be a cost-effective way of implementing an EHIS in these resource-limited services. However the extent to which an EHIS delivered using a mobile technology platform is feasible to implement and acceptable to treatment providers needs to be fully explored before such a system is developed. This is important given that several participants had privacy, confidentiality and legal concerns about using a central EHIS shared across the substance abuse treatment system. Since a common information system and a single set of records would allow for the performance of facilities to be compared and result in more efficient information gathering [44], it will be important to identify ways in which these concerns can be resolved, such as utilising advances in information technology to safeguard sensitive health information.

Participants also cautioned against developing a performance measurement system without collaborating with service providers to ensure that the system is responsive to their needs and constraints. They advised that any new system imposed on them without appropriate consultation was likely to be met with resistance. This is not altogether surprising as evidence from other studies suggests that previous top-down attempts to impose change on the South African substance abuse treatment system failed because providers refused to adopt the new systems as they had not been properly consulted about the new initiatives [5,36]. Furthermore, where performance measurements have been successfully implemented in health care settings elsewhere, implementation has been characterised by supportive organisational environments where health care personnel have been invested in service monitoring and quality assurance processes [39,40]. Together these findings highlight the importance of embracing a participatory approach to measuring the performance of South African substance abuse treatment services in which treatment providers are treated as partners in the developmental process rather than as passive recipients of the system. This participatory approach may increase providers’ propensity to implement the system [45] and the likelihood of this system generating information that is useful to providers [46].

Finally, findings indicate that in order for the proposed performance measurement system to produce data that will be used to improve the quality of substance abuse treatment, service managers will need to be trained to understand facility-level data generated by this system. For the most part, participants in this study produced data they were unable to use to inform practice because they lacked the requisite skills to analyse and interpret data. This is similar to experiences from the US where providers’ lack of research and evaluation-related skills limited the extent to which they used research findings to improve practice [46]. On a positive note, participants in this study were eager to be trained in how to understand and use their own data to improve practice. Future implementation of a performance measurement system therefore is likely to benefit from capacity-building initiatives that give service managers the tools needed to generate simple reports about their facilities’ performance on key indicators. Mobile technologies and advances in information technology (such as the use of webinars and the provision of free access to e-tools that service managers can use to generate simple reports) provide affordable alternatives to in-person training that are likely to be feasible to implement in South Africa.

Findings from this study should be considered in the light of two main limitations. As this qualitative study was limited to treatment providers from two of the nine provinces in South Africa, findings may not be generalisable to providers from other provinces. However, as our sample included treatment providers across the entire spectrum of facilities available in the country (including poorly and better resourced services and programs of varying intensity) we are relatively confident that the issues and recommendations raised by our participants will be broadly applicable to all substance abuse treatment providers in South Africa. Second, our study design meant that we were unable to explore whether there were within-facility differences in attitudes and perceptions towards treatment service quality monitoring. It is possible that clinical staff and treatment directors may have had divergent views on the feasibility and potential value of a performance measurement system for substance abuse treatment. Further studies are needed to compare and contrast clinical staff and treatment directors’ views towards performance measurement. As the participation of treatment providers is a prerequisite for the successful implementation of any performance measurement system [27], any concerns will need to be addressed prior to the implementation of such a system.

## Conclusion

Limitations notwithstanding, this exploratory study shows that South African substance abuse treatment providers are willing to adopt a performance measurement system for substance abuse treatment that uses scientifically robust tools to collect data on a small set of quality indicators. Findings also suggest several actions that need to be taken to ensure that this performance measurement system fulfils its promise of collecting data that can be used to identify targets for quality improvement. First, the development of this system has to occur in collaboration with treatment providers to ensure that it is responsive to their needs and challenges. Second, a core principle of this proposed system should be minimising burden on treatment providers by only collecting data on a core set of shared indicators. Related to this, if a decision is made to develop an EHIS to facilitate performance measurement, the feasibility and acceptability of using mobile health applications should be explored to limit the resource demands associated with EHIS and the introduction of an EHIS should be accompanied by appropriate infrastructure support. Third, the implementation of the proposed system will need to be accompanied by capacity building and support for service providers, at least in the initial stages of implementation, so that providers are empowered to use their data for its intended purpose.

## Abbreviations

AOD: Alcohol and other drug; EHIS: Electronic health information system.

## Competing interests

The authors declare that they have no competing interests.

## Authors’ contributions

BM was the principal investigator on the study and was responsible for study design, planning, writing and reviewing all aspects of the manuscript. ZP and RK conducted the focus groups and analyses with inputs from BM and assisted with writing the methods and results of the first draft of the manuscript. ZP, RK, JRK, RM, JB and CP all commented on the first draft or the manuscript and read and approved the final draft.

## Pre-publication history

The pre-publication history for this paper can be accessed here:

http://www.biomedcentral.com/1471-244X/14/31/prepub

## Supplementary Material

Additional file 1 Focus group protocol for service providers. The SQM Project. Click here for file
