# Supplementary material for: Identifying perceived barriers to monitoring service quality among substance abuse treatment providers in South Africa
Source: BMC Psychiatry. 2014 Feb 5;14:31. doi: 10.1186/1471-244X-14-31 (PMC3917424; doi:10.1186/1471-244X-14-31)
Supplement: Additional file 1 — Focus group protocol for service providers. The SQM Project. [file 1471-244X-14-31-S1.doc]

1. **How do you currently monitor the quality of services you provide in this facility?**

**Prompts:**

- (e.g., number of clients served from SACENDU, treatment process indicators, outcome studies)
- Is there uniformity in how you monitor your services and how other facilities monitor their services
- What do you do with the data you collect on service quality?

1. **In your opinion, what are the main barriers to monitoring the quality of treatment in your facility?**

**Prompts:**

- Gaps in type of data collected?
- Gaps in how data are stored?
- Resources?
- Staff investment in data collection?
- Other?

1. **In your opinion what is needed to facilitate the collection of data on service quality? Why?**

**Prompts:**

- Resources?
- Training?
- Support?
- Other?

1. **Describe proposed performance measurement system to participants. In principle would you be willing to adopt this system for measuring service quality?**

- Why?
- Do you have any concerns about the proposed system?
